# Heschl’s gyrus duplication pattern and clinical characteristics in borderline personality disorder: A preliminary study

**DOI:** 10.3389/fpsyt.2022.1033918

**Published:** 2022-11-03

**Authors:** Tsutomu Takahashi, Daiki Sasabayashi, Dennis Velakoulis, Michio Suzuki, Patrick D. McGorry, Christos Pantelis, Andrew M. Chanen

**Affiliations:** ^1^Department of Neuropsychiatry, University of Toyama Graduate School of Medicine and Pharmaceutical Sciences, Toyama, Japan; ^2^Research Center for Idling Brain Science, University of Toyama, Toyama, Japan; ^3^Department of Psychiatry, Melbourne Neuropsychiatry Centre, The University of Melbourne and Melbourne Health, Carlton, VIC, Australia; ^4^Neuropsychiatry Unit, Royal Melbourne Hospital, Melbourne Health, Melbourne, VIC, Australia; ^5^Orygen, Melbourne, VIC, Australia; ^6^Centre for Youth Mental Health, The University of Melbourne, Melbourne, VIC, Australia; ^7^Florey Institute of Neuroscience and Mental Health, Parkville, VIC, Australia; ^8^North Western Mental Health, Western Hospital Sunshine, St Albans, VIC, Australia

**Keywords:** superior temporal gyrus, gyrification, sulco-gyral pattern, externalizing behavior, disruptive behavior disorders, magnetic resonance imaging

## Abstract

Inter-individual variations in the sulco-gyral pattern of Heschl’s gyrus (HG) might contribute to emotional processing. However, it remains largely unknown whether borderline personality disorder (BPD) patients exhibit an altered HG gyrification pattern, compared with healthy individuals, and whether such a brain morphological feature, if present, might contribute to their clinical characteristics. The present study used magnetic resonance imaging to investigate the distribution of HG gyrification patterns (single or duplicated) and their relationship to clinical characteristics in teenage BPD patients with minimal treatment exposure. No significant difference was noted for the prevalence of HG patterns between 20 BPD and 20 healthy participants. However, the BPD participants with left duplicated HG were characterized by higher prevalence of comorbid disruptive behavior disorders, with higher externalizing score compared with those with left single HG. Our preliminary results suggest that neurodevelopmental pathology associated with gyral formation might be implicated in the neurobiology of early BPD, especially for emotional and behavioral control.

## Introduction

Heschl’s gyrus (HG), which is a convolution on the surface of superior temporal gyrus (STG), contains the primary auditory cortex and is central to auditory processing (1), while also having a prominent role in emotional information processing (2, 3). HG is known for its high inter-individual anatomical variability, potentially due to variations in cytoarchitectonic development during fetal life; about 30–50% of healthy adults have a partial split of the lateral part of the gyrus (i.e., partial duplication) or independent two gyri (complete duplication) (4, 5). Although the functional significance of different HG patterns remains unclear, HG duplication might be related to decreased HG activity during auditory processing (6) and learning impairment (7, 8). It is also reported that hyper-gyrification (i.e., extensive cortical folding) in the STG region is weakly associated with motor impulsivity (9) and irritability (10) in healthy young adults. However, the role of a HG duplication pattern on personality traits characterized by emotional dysregulation remains unknown.

Although the neurobiology of borderline personality disorder (BPD) has yet to be elucidated, abnormalities in neural networks, including the STG, have been implicated in their impulsive behaviors and emotional instability (11–13). Furthermore, previous magnetic resonance imaging (MRI) studies have reported that BPD patients exhibit brain morphological characteristics associated with fetal neurodevelopmental abnormalities (e.g., altered sulco-gyral patterns and hyper- or hypo-gyrification) at early stages of the illness (14–18). While our previous MRI study found no volume changes of HG in a BPD cohort and its clinical subgroups (e.g., with and without violent episodes) (19), no MRI studies have specifically investigated HG duplication patterns in BPD.

This MRI study examined the distribution of HG gyrification patterns in BPD teenagers who had received minimal treatment and in healthy control participants. Based on a possible role for the STG in emotional dysregulation in BPD (11) and structure-function relationships of HG gyrification patterns (6), we predicted that BPD patients would have an altered prevalence of HG duplication. We also explored whether the HG gyrification pattern was related to BPD phenomenology.

## Materials and methods

### Participants

The present study included 20 teenagers with BPD and 20 healthy controls (Table 1). Recruitment strategy and sample characteristics of this cohort have been detailed elsewhere (20). All participants in this study had no history of significant medical problems that could affect brain function and/or mental conditions (e.g., thyroid diseases, serious brain injury, seizure, neurological illness, or other).

**TABLE 1:** Demographic and clinical characteristics of the study participants.

Variable	Healthy controls	BPD patients	Group comparison
Age (years)	19 ± 2.2	17.3 ± 1.1	ANOVA: *F* (1, 38) = 2.97, *p* = 0.003
	(range, 16.2–23.8)	(range, 15.4–19.2)	
Gender (males/females)	5/15	5/15	χ^2^ = 0.00, *p* = 1.000
Hand dominance (right/left/mixed)	18/2/0	18/1/1	Fisher’s exact test: *p* = 0.513
NART-estimated IQ^a^	101.9 ± 9.1	100.9 ± 5.8	ANOVA: *F* (1, 37) = 0.20, *p* = 0.654
Life time trauma exposure (yes/no)^b^	–	10/9	
Parasuicidal episodes in 6 months (yes/no)	–	13/7	
Number of parasuicidal episode	–	11.0 ± 9.8 (*N* = 13)	–
Violent episodes in 6 months (yes/no)	–	9/11	
Number of violent episode	–	6.4 ± 9.2 (*N* = 9)	–
YASR or YSR subscale scores			
Internalizing	–	0.91 ± 0.49	–
Externalizing	–	0.76 ± 0.41	–
SCID-II total BPD score	–	21 ± 3.2	

Values represent means ± SDs. ANOVA, analysis of variance; BPD, borderline personality disorder; NART, National Adult Reading Test; SCID-II, Structured Clinical Interview for DSM-IV Axis II Disorders; YASR, Young Adult Self-Report; YSR, Youth Self-Report. ^*a*^Data were not available for some participants. ^*b*^Interview data were not available for one BPD patient.

Briefly, BPD teenagers meeting the Structured Clinical Interview for DSM-IV Axis II Disorders (SCID-II) criteria (21) but who had never received specific treatment for BPD, were recruited from the Helping Young People Early (HYPE) Clinic, an early intervention service for BPD in Melbourne, Australia (22). Major comorbid Axis I diagnoses were: disruptive behavior disorders (*N* = 10), mood disorder (*N* = 7), anxiety disorder (*N* = 9), and/or substance use disorders (*N* = 6). They were medication-free at scanning except for three patients who had received antidepressants. At intake, they were assessed for lifetime trauma exposure (physical, emotional, and/or sexual) and parasuicidal/violent episodes (Table 1) *via* a semi-structured interview.

The patients also completed the Young Adult Self-Report [YASR (23)] (age ≥18 years) or the Youth Self-Report [YSR (24)] (age <18 years).

Healthy comparison subjects were selected from a database of healthy volunteers who had no personal of family history of psychiatric disorders or substance abuse/dependence. The SCID-II derived checklist was used to confirm that they did not have any BPD symptoms. This study was approved by Melbourne Health Mental Health Research and Ethics Committee (MHREC2009.607). In accordance with the Declaration of Helsinki, Study participants or a parent or guardian gave written informed consent, prior to participating in the study.

### Magnetic resonance imaging procedures

Magnetic resonance images were obtained using a 1.5T GE Signa scanner, with a three-dimensional volumetric spoiled gradient recalled echo sequence to provide 124 contiguous coronal slices of 1.5 mm thickness. Detailed imaging parameters were described elsewhere (17, 19).

**FIGURE 1:**
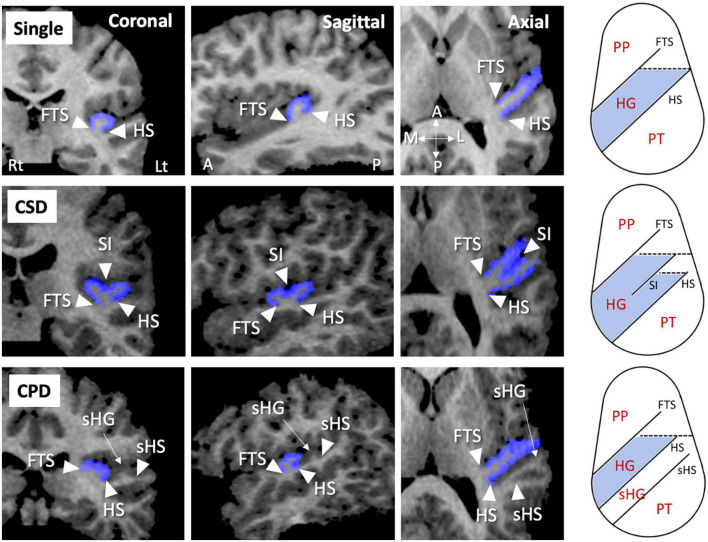
Sample MR images of different gyrification pattern in the Heschl’s gyrus (HG) (colored in blue). These HG patterns have been demonstrated also in our previous publications (22–26). A, anterior; CPD, complete posterior duplication; CSD, common stem duplication; FTS, first transverse sulcus; HS, Heschl’s sulcus; L, lateral; Lt, left; P, posterior; M, medial; PP, planum polare; Rt, right; sHG, second Heschl’s gyrus; sHS, second Heschl’s sulcus; SI, sulcus intermedius.

As fully described previously (25–29), the HG gyrification patterns were classified into single or duplicated patterns on the reformatted MR images (i.e., 0.938 mm iso-voxel images) using Dr. View (Infocom, Tokyo, Japan); the duplicated HG patterns were subdivided into partial [i.e., common stem duplication (CSD)] or complete [i.e., complete posterior duplication (CPD)] patterns (Figure 1). All HG gyrification patterns were classified by one rater (TT) with no knowledge of the subjects’ identities. A validation study of HG pattern classification in a randomly selected 20 hemispheres showed sufficient inter- (TT and DS) and intra-rater (TT) reliabilities (Cronbach’s α > 0.80).

### Statistical analysis

Group differences in the HG pattern distribution (single, CSD, or CPD) were compared for each hemisphere using the χ^2^ test or Fisher’s exact test. Given that only four hemispheres in the BPD group had the CPD pattern (Table 2) and that partial and complete duplications likely have no differences in tonotopic organization of human auditory cortex (30), similar to a previous study examining the relationship between the HG patterns and HG activity (6), the CSD and CPD patterns were categorized together as “duplicated pattern” for subsequent analyses. Relationships between the HG gyrification patterns and BPD subgroups (i.e., with or without the trauma exposure, violent/parasuicidal behaviors, and comorbid DSM diagnoses) were also assessed by the χ^2^ test or Fisher’s exact test. Because of the small sample size, the non-parametric Mann–Whitney U test was used to evaluate the potential contribution of HG gyrification pattern to clinical variables (IQ, YSR/YASR subscale scores, number of suicidal and violent episodes, and SCID-II total BPD score). Statistical significance was set at *p* < 0.05.

**TABLE 2:** Gyrification pattern of Heschl’s gyrus (HG) in the study participants.

Healthy controls					
		**Right HG pattern [*N* (%)]**			
		**Single**	**CSD**	**CPD**	**Total**
Left HG pattern [*N* (%)]	Single	6 (30.0)	6 (30.0)	2 (10.0)	14 (70.0)
	CSD	2 (10.0)	2 (10.0)	1 (5.0)	5 (25.0)
	CPD	1 (5.0)	0 (0.0)	0 (0.0)	1 (5.0)
	Total	9 (45.0)	8 (40.0)	3 (15.0)	20 (100.0)

**BPD**					

		**Right HG pattern [*N* (%)]**			
		**Single**	**CSD**	**CPD**	**Total**

Left HG pattern [*N* (%)]	Single	5 (25.0)	5 (25.0)	0 (0.0)	10 (50.0)
	CSD	3 (15.0)	3 (15.0)	3 (15.0)	9 (45.0)
	CPD	0 (0)	1 (5.0)	0 (0.0)	1 (5.0)
	Total	8 (40.0)	9 (45.0)	3 (15.0)	20 (100.0)

CSD, common stem duplication; CPD, complete posterior duplication.

## Results

### Sample characteristics

There were no significant group differences in gender ratio, height, handedness, and IQ, while BPD patients were younger than controls (Table 1). The BPD participants with comorbid disruptive behavior disorders had a higher YSR/YASR externalizing score (*N* = 10; mean = 1.04, SD = 0.37) than those without (*N* = 10; mean = 0.49, SD = 0.22) [*F* (1, 18) = 17.13], *p* < 0.001], but other demographic and clinical variables did not differ between these subgroups.

### Heschl’s gyrus pattern distributions

**FIGURE 2:**
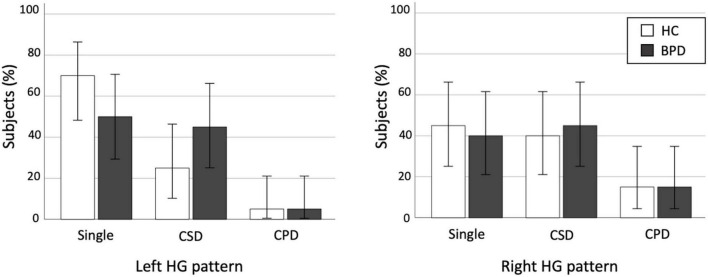
Distribution of Heschl’s gyrus (HG) duplication patterns in the healthy control (HC) and borderline personality disorder (BPD) groups. CPD, complete posterior duplication; CSD, common stem duplication. Error bars show 95% confidence intervals.

We found no significant differences in the prevalence of HG patterns bilaterally between the BPD and control groups (Table 2 and Figure 2), even when the CSD and CPD patterns were categorized together as the duplicated pattern (all *p* > 0.197).

### Association between the Heschl’s gyrus pattern and demographic/clinical characteristics

Gender and IQ were not related to the HG gyrification pattern for both BPD and healthy control groups.

**FIGURE 3:**
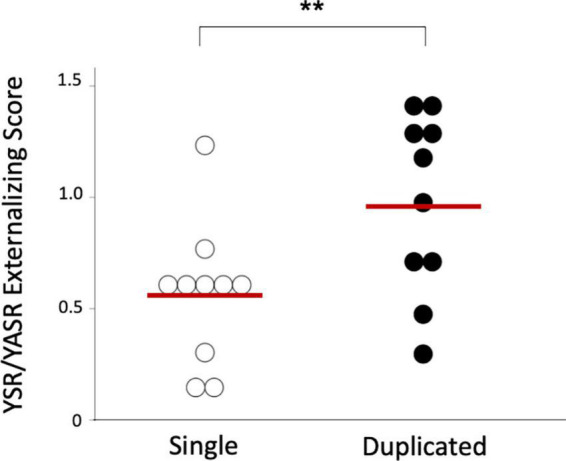
Youth Self-Report (YSR) or Young Adult Self-Report (YASR) externalizing scores in the borderline personality disorder patients with single and duplicated Heschl’s gyrus patterns on the left hemisphere. Horizontal bars indicate means of each group. *p < 0.05.

The BPD patients with left duplicated HG were characterized by higher YSR/YASR externalizing scores (*U* = 79.0, *p* = 0.029) (Figure 3) and higher prevalence of comorbid disruptive behavior disorders (Fisher’s exact test, *p* = 0.023) (Table 3), compared with those with left single HG. Other clinical variables and subgroups of the BPD patients were not related to HG gyrification patterns.

**TABLE 3:** Gyrification pattern of Heschl’s gyrus (HG) in borderline personality disorder (BPD) patients with and without comorbid disruptive behavior disorders.

BPD with disruptive behavior disorders				
		**Right HG pattern [*N* (%)]**		
		**Single**	**Duplicated**	**Total**
Left HG pattern [*N* (%)]	Single	1 (10.0)	1 (10.0)	2 (20.0)
	Duplicated	3 (30.0)	5 (50.0)	8 (80.0)
	Total	4 (40.0)	6 (60.0)	10 (100.0)

**BPD without disruptive behavior disorders**				

		**Right HG pattern [*N* (%)]**		
		**Single**	**Duplicated**	**Total**

Left HG pattern [*N* (%)]	Single	4 (40.0)	4 (40.0)	8 (80.0)
	Duplicated	0 (0.0)	2 (10.0)	2 (20.0)
	Total	4 (40.0)	6 (60.0)	10 (100.0)

## Discussion

To our knowledge, this is the first study examining the HG duplication pattern and its relationship to clinical characteristics in BPD. While the prevalence of HG duplication did not differ between the adolescent BPD patients and control subjects, the patients who had a duplicated HG on the left hemisphere were characterized by more severe aggressive behavior, compared with those with a single HG. The present results suggest that neurodevelopmental characteristics associated with fetal gyral formation might contribute to clinical subtypes and/or symptom severity early in the course of BPD.

Previous MRI studies in adolescent BPD demonstrated gray matter reduction and/or significant relationship with aggression/impulsivity predominantly in fronto-limbic brain regions (31, 32), which could not be explained by confounding factors associated with illness chronicity and treatment (33). Interestingly, a few diffusion tensor imaging studies in adolescent BPD (34–36) supported the notion that abnormalities in fronto-limbic networks are associated with emotional dysregulation and impulsivity early in the course of BPD (33). Further, recent MRI findings of gross anatomical features in BPD patients [e.g., altered cortical surface morphology (15, 17, 18)], which reflect prenatal brain development (37), may at least partly support their early neurodevelopmental pathology (33). However, there are discrepancies in previous cortical folding findings in BPD; Vatheuer et al. (18) demonstrated a parietal hyper-gyrification, while Depping et al. (15) reported a significant relationship between hypo-gyrification of the orbitofrontal region and impulsivity. Thus, potential role of early neurodevelopmental processes associated with cortical folding on the pathophysiology of BPD may have regional specificity.

The present findings of HG duplication pattern appear to reflect fetal neurodevelopment, because variations in the HG gyrification pattern are formed largely during the late gestation period along with neural development (38, 39) but remain rather stable after birth (40). Despite the small sample size, the prevalence and pattern (i.e., more frequent in right hemisphere) of HG duplication in our healthy subjects were comparable with previous reports in large samples (4, 5, 41). The BPD group was characterized by higher prevalence of HG duplication on left hemisphere (50%) than healthy subjects (30%) [odds ratio = 2.33 (95% CI, 0.64–8.54)] with small-to-medium effect size (*Phi* = 0.204), but this difference was not statistically significant. This negative result might reflect the heterogeneity of the disorder (42), because the HG patterns were associated with specific subtypes and symptoms in our BPD cohort as detailed below. It is reported that antipsychotic medication (43) and adverse environmental factors [e.g., childhood maltreatment (44)] might also affect gyrification in the adult brain, but we found no effects of trauma exposure on HG patterns in our BPD cohort with minimal treatment exposure.

In the present study, our results suggested that left duplicated HG in BPD might contribute to higher score for delinquent and aggressive behavior (i.e., externalizing score) and higher prevalence of comorbid disruptive behavior disorders, supporting the notion that BPD is a heterogeneous disorder with different neurobiological underpinnings for core endophenotypes, such as emotional dysregulation and impulsive aggression (45). Although the present study cannot directly address the functional significance of HG patterns in the neurobiology of BPD, our results are consistent with previous findings that the HG duplication is associated with impaired HG functioning (6) and that abnormal neural networks including the STG contribute to impulsive behaviors and emotional instability in BPD (11–13). These findings seem to support the early neurodevelopmental model of BPD that neurobiological vulnerability associated with fetal sulcal formation might contribute to specific clinical characteristics in early stages of BPD. However, further studies will be needed to clarify the role of environmental factors after birth that might further increase the risk of BPD in vulnerable individuals (33, 46).

There are several potential confounding factors in this study. First, the present study was clearly limited by a lack of statistical power to reliably detect group differences due to small sample size. Because the BPD group in this study had a somewhat higher prevalence of left HG duplication compared to controls (Table 2 and Figure 2), the possibility exists that future investigation in a larger BPD cohort might be able to detect significant group differences. Similarly, potential gender differences in brain gyrification (47) could not be reliably examined in our small sample especially for male subjects. Second, younger age of the BPD patients (mean = 17.3 years), compared with control participants (mean = 19.0 years) in this study, might have biased our results. However, it is unlikely that this difference in a narrow age would have a major impact on gross sulco-gyral pattern, which is a rather stable neurodevelopmental marker (40). Indeed, we found no effect of age on the HG patterns (single vs. duplicated) in the present sample [left, *F* (1, 38) = 1.02, *p* = 0.320; right, *F* (1, 38) = 0.12, *p* = 0.744]. Furthermore, the effect of age alone could not explain our main finding of different prevalence of HG duplication between the BPD subgroups (with and without disruptive behavior disorders), since these subgroups did not differ for age. Third, the CSD and CPD patterns were categorized together in this study because only a few hemispheres had the CPD pattern. While the functional role of the HG duplication type (i.e., CPD vs. CSD) remains largely unknown, our previous study in schizophrenia suggested specific role of the CSD pattern on cognitive deficits (27). Thus, future studies should examine whether different HG duplication patterns play different roles in the pathophysiology of BPD. Finally, the present study cannot address whether the relationship between the HG duplication and emotional/behavioral characteristics is specific to BPD, because of the lack of a clinical comparison group and because the healthy participants in this study were not comprehensively assessed for personality pathology or behavioral characteristics. It remains unanswered whether participants with disruptive behavior disorders, but without BPD features, have an altered HG pattern. Further, we have previously demonstrated the association between HG duplication and “lack” of emotional responsivity in schizophrenia (28), suggesting different contribution of HG patterns on clinical characteristics in different disorders/conditions. It should be noted that we examined only the HG patterns, but not other biological features, and their relationship with clinical and behavioral feature of our BPD cohort. Thus, the disease specificity of our HG findings and their functional significance should be further tested using larger samples of various clinical/non-clinical populations.

In summary, our preliminary results demonstrated a relationship between the HG duplication pattern and BPD phenomenology (especially aggressive behavior) in teenagers with first-presentation BPD. Thus, neurobiological vulnerability associated with fetal sulcal formation might increase the risk for impaired control of emotion and behavior in the early stages of BPD.

## Data availability statement

The raw data supporting the conclusions of this article will be made available by the authors, without undue reservation.

## Ethics statement

The studies involving human participants were reviewed and approved by the Melbourne Health Mental Health Research and Ethics Committee. Written informed consent to participate in this study was provided by the participants’ legal guardian/next of kin.

## Author contributions

AC, MS, CP, and PM conceived the idea and methodology of the study. TT conducted the statistical analyses and wrote the manuscript. AC and DV recruited subjects and involved in clinical and diagnostic assessments. TT and DS analyzed the MRI data. AC, CP, and MS contributed to the writing and editing of the manuscript. All authors contributed to the article and approved the submitted version.
